# Supplementary material for: B cell zone reticular cell microenvironments shape CXCL13 gradient formation
Source: Nat Commun. 2020 Jul 22;11:3677. doi: 10.1038/s41467-020-17135-2 (PMC7376062; doi:10.1038/s41467-020-17135-2)
Supplement: Supplementary file 1 — Supplementary Information [file 41467_2020_17135_MOESM1_ESM.pdf]

Cosgrove et al., B-cell Zone Reticular Cell Microenvironments Shape CXCL13 Gradient Formation

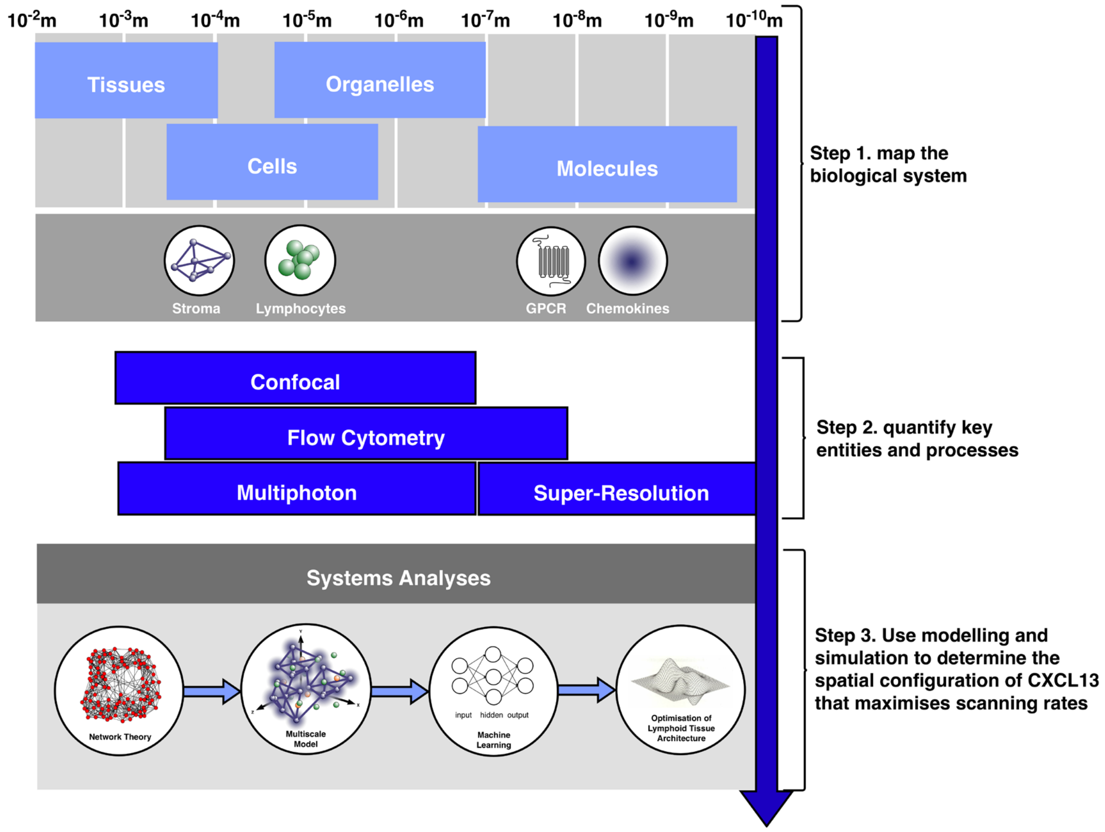

Supplementary Figure 1. CXCL13 is a key determinant of lymphoid tissue structure, and antibody mediated immune responses. CXCL13 is subject to multiple simultaneously occurring regulatory mechanisms that act over broad spatiotemporal scales. Consequently, an ensemble of imaging and systems biology approaches are required to understand this phenomenon. Using systems-based techniques we develop a conceptual model of the system and quantify key entities using an ensemble of imaging approaches. This then informs development of a multiscale model to consolidate data across multiple levels of biological organization concurrently. Machine learning techniques can be then used to emulate the multiscale model; in reducing the computational expense of the simulator it is then possible to perform analyses such as multi-objective optimization to determine the Pareto optimal (as defined in the materials and methods) spatial distribution of CXCL13 within the primary follicle.

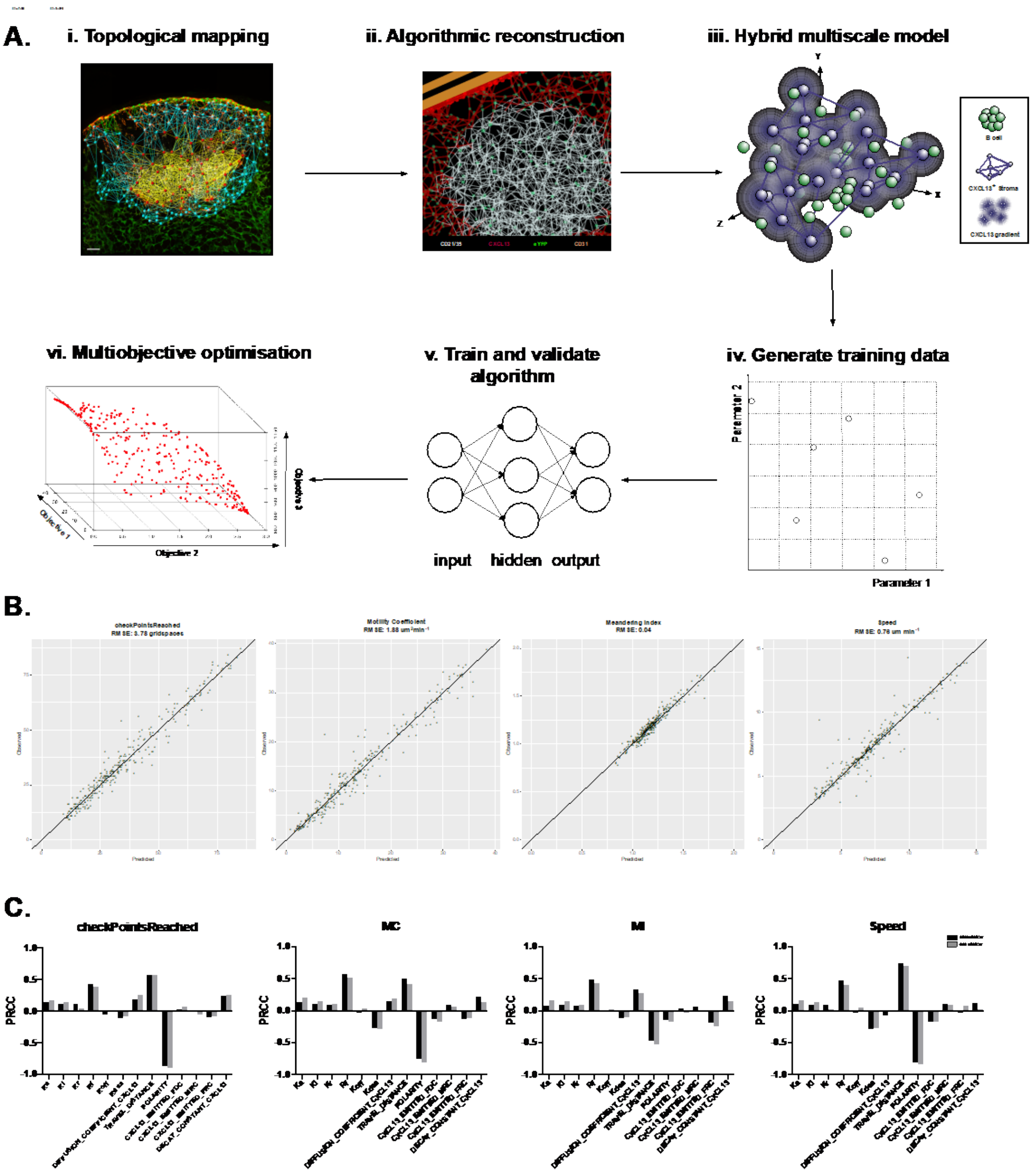

Supplementary Figure 2. Overview of the emulation pipeline and predictive performance of the emulator. In this scheme key topological parameters (i) described in Figure 2 are used to inform an algorithmic reconstruction of the CXCL13^{+} follicular stromal cell network (ii). This network is then integrated into a hybrid multiscale model where in silico B cells migrate in response to CXCL13 gradients created by the *in silico* stromal cell network (iii). We summarize the behaviors of the simulator using a Latin hypercube sampline approach (iv) and use this data to train a neural network to provide a mapping function between parameter inputs and emergent cellular behaviors (v). The emulator then acts as a surrogate tool for the simulator, capable of converting a set of parameter values into a prediction of the simulation response that is representative of a high number of replicates. In saving resources, emulation can serve as a useful adjunct to the original simulator, providing insights where complex analyses were previously intractable such as permitting use of multi-objective evolutionary algorithms (vi) as described in Figure 3. (B) Benchmarking the performance of the emulator in terms of its ability to predict simulator outputs, and also to quantify parametric uncertainty using the partial rank correlation coefficient in conjunction with a global perturbation approach (C). Source data are provided as a Source Data file.

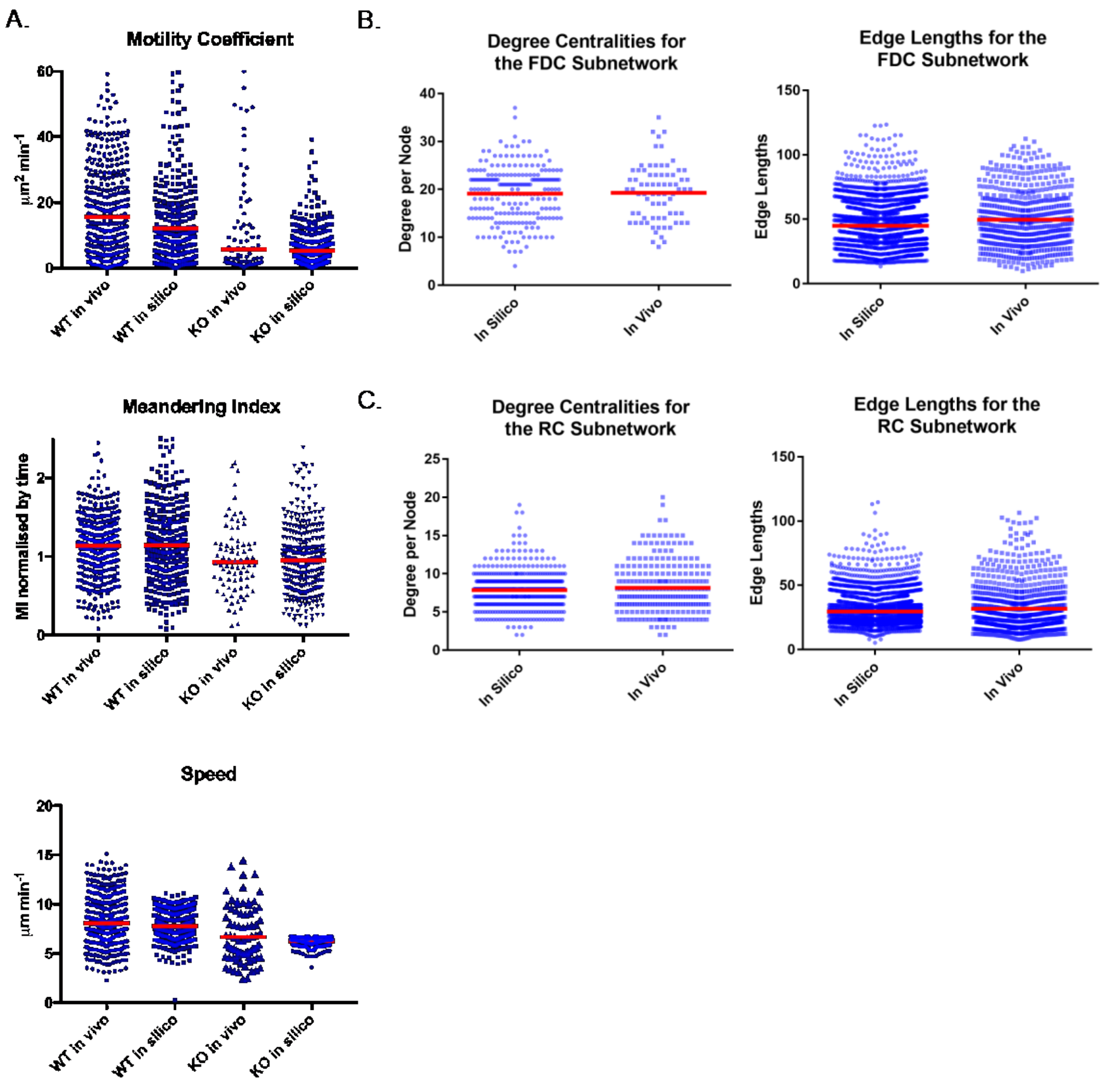

Supplementary Figure 3. Calibration of emergent behaviors against experimental behaviors. (A)The migration patterns of B cells were measured by injecting 5 x 10^6 purified B cells from C57BL/6 (wild type (WT)) or CXCR5 deficient mice labeled with the fluorescent cell staining dyes Carboxyfluorescein succinimidyl ester and CellTracker Orange (5-(and-6)-(((4-chloromethyl)benzoyl)amino)tetramethylrhodamine) CMTMR into age- and sex-matched wild type mice and imaged with two-photon microscopy [1]. All datasets were non-normal as determined by a Shapiro-Wilk test, subsequently; a Mann-Whitney test was used to determine if datasets were significantly different at a significance level of 5%. Bar charts represent the median value with error bars representing the I.Q.R. (B-C) Comparison of *in vivo* and *in silico* distributions of degree centrality and edge length values for stromal cell subsets. No statistically significant differences were found between the median values for *in silico* and *in vivo* degree centralities or edge lengths for the global network or associated subnetworks. Significance assessed using a Mann-Whitney test with $p < 0.05$ representing a statistically significant result. Source data are provided as a Source Data file.

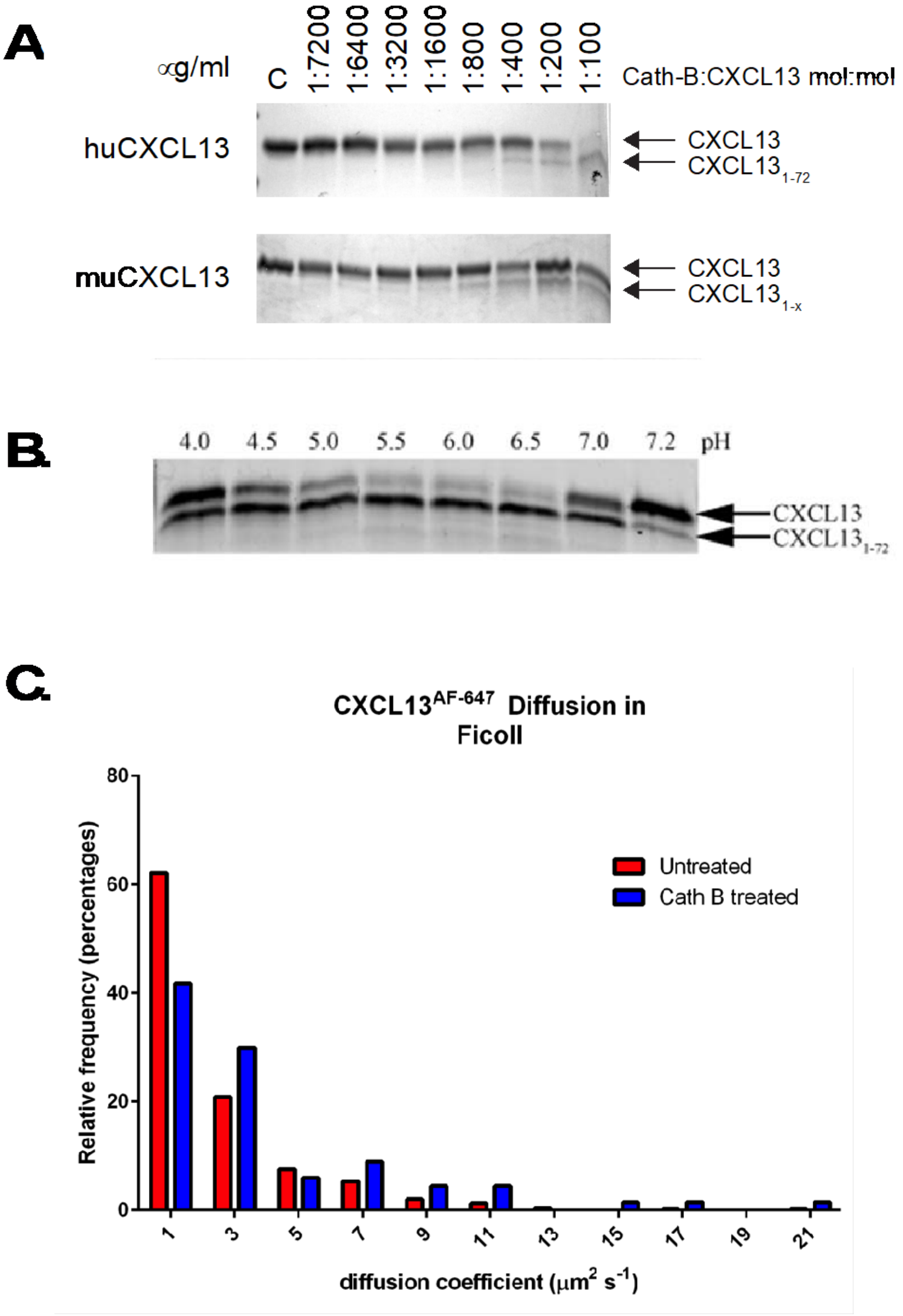

Supplementary Figure 4. Cathepsin B mediated cleavage of CXCL13. (A) Human (upper panel) and mouse (lower panel) 8.3 μM CXCL13 were incubated for 4h with increasing amounts of either human of mouse Cath-B ranging from 0.34 to 86 nM. c = intact CXCL13 (B) 72 nM human Cath-B was incubated with 4 μM CXCL13 at the indicated pH values. The cleavage products were separated by SDS-PAGE and stained with Coomassie blue. (C) Comparison of $CXCL13^{AF647}$ microdiffusion coefficient distributions in 15% Ficoll. Distributions of CXCL13 microdiffusion coefficients in tonsils treated with 72nM of Cathepsin B for 1 hour at 37C are shown in blue with untreated control samples shown in red. Source data are provided as a Source Data file.

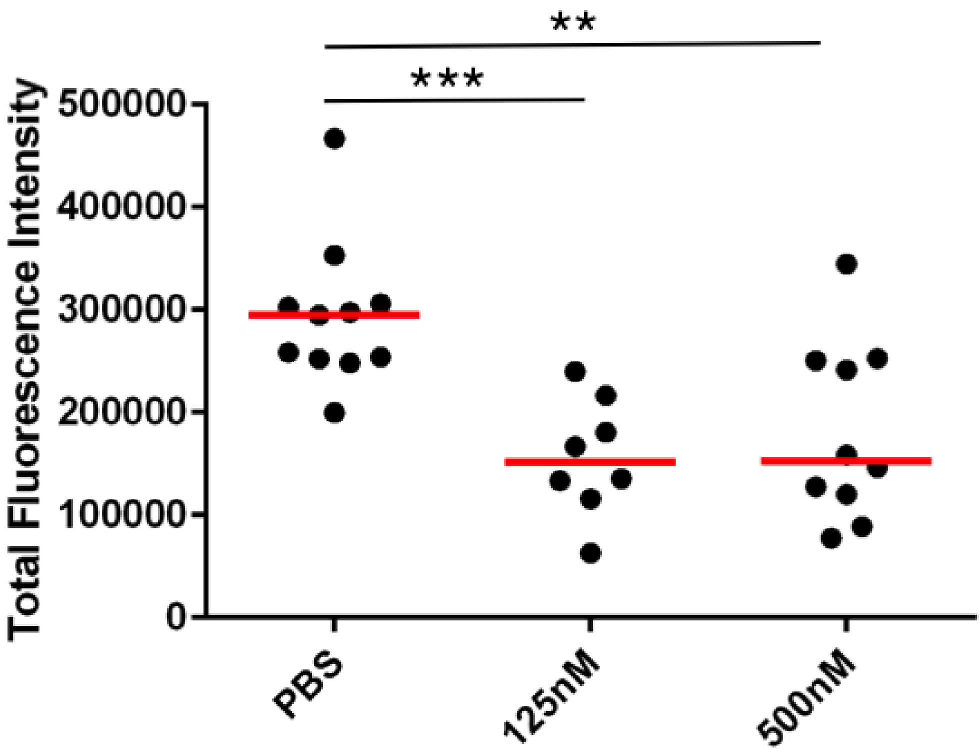

Supplementary Figure 5. Tonsil tissue sections were incubated in PBS or Cathepsin B (CathB) (at the concentration stated) and stained with antibodies against CD19 and CXCL13. The total fluorescent intensity of CXCL13 signal in each fixed size image was then quantified to facilitate comparison. Significance was assessed using a Students T-test. ** $p < 0.01$, *** $p < 0.001$. Source data are provided as a Source Data file

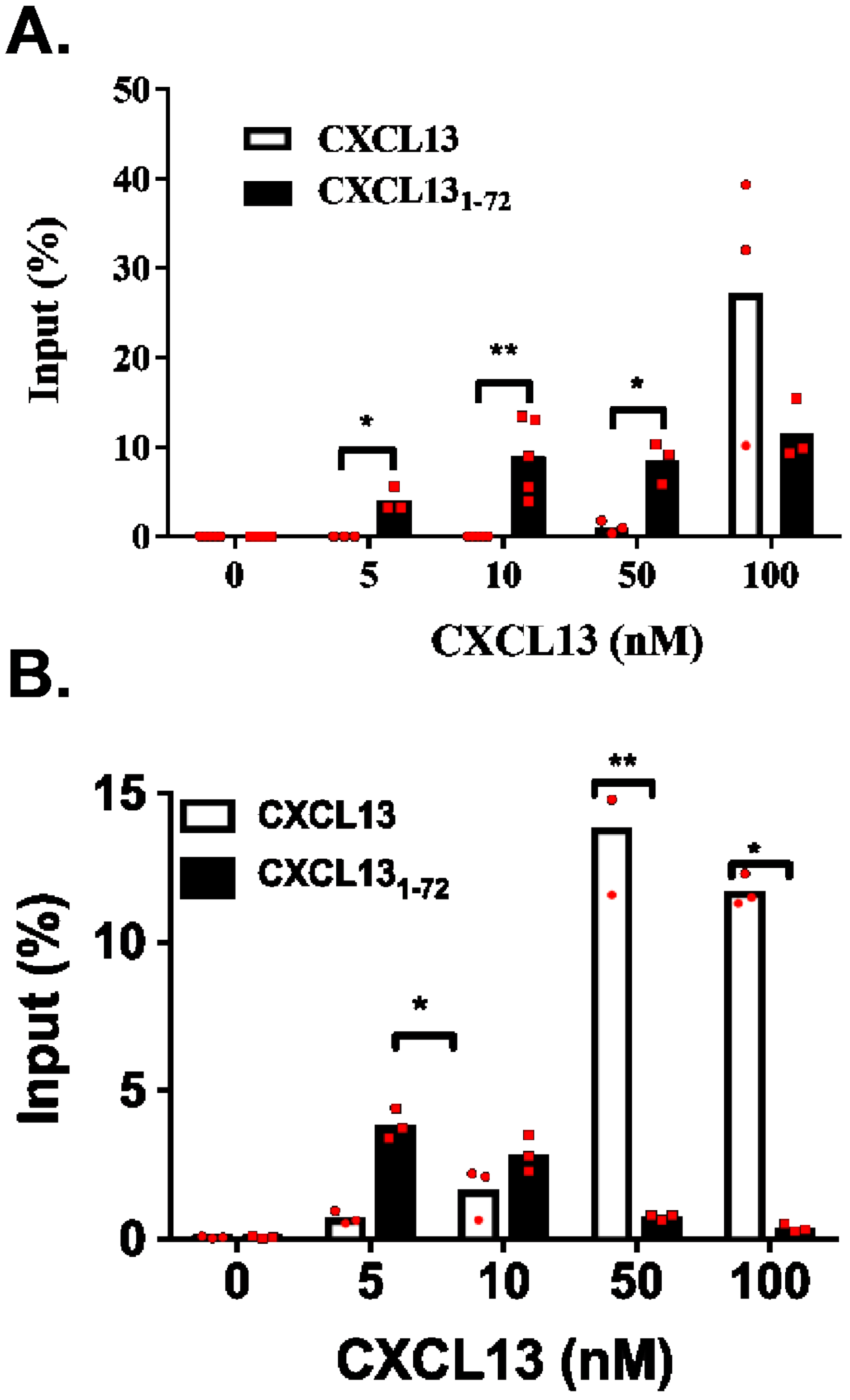

Supplementary Figure 6. Cell migration in response to intact and truncated CXCL13 was evaluated in 2D and 3D. (A) Chemotaxis of CXCR5-transfected Pre-B 300-19 cells using 5 μm pore size transwell filters. (B) 3-dimensional migration of CXCR5-transfected Pre-B 300-19 cells in Matrigel. Data represents the percentage of migrated cells relative to the number of cells added to the transwell filters. Values (mean ± SD) represent at least three independent experiments. Statistically significant differences are indicated, *$p < 0.05$ and ** $p < 0.01$. Significance, was assessed using a Students T-test. Source data are provided as a Source Data file

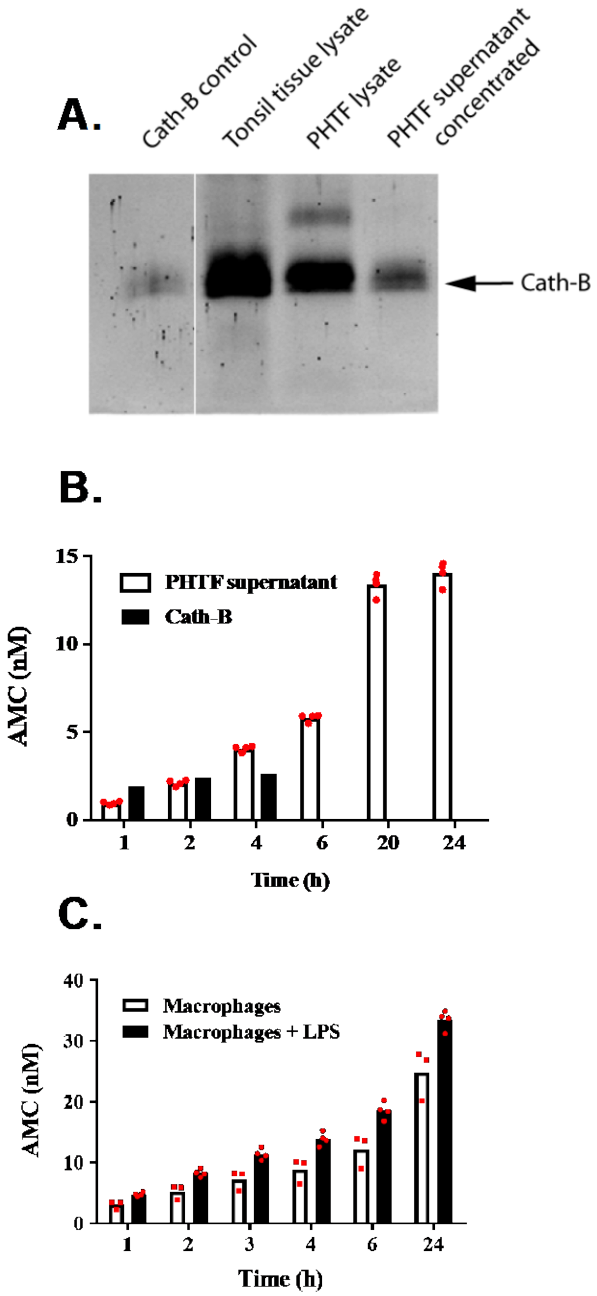

Supplementary Figure 7. Cath-B expression in tonsil fibroblasts (A) Immunoblots for Cath-B detection was performed with 25µg protein equivalents from lysates of tonsil tissue and PHTFs and from concentrated supernatants of cultivated PHTFs. Purified Cath-B (5 ng) from human liver was used as a control. (B) Cath-B activity in PHTF supernatants was analyzed using Z-Arg Arg AMC as substrate. 1.2ng purified Cath-B from human liver was used as control. Data show mean (± SD) of at least four independent experiments carried out in duplicates. (C) Monocyte-derived macrophages in 12 well microtiter plates were incubated with or without 1µg/ml LPS for 3 h. Supernatant was collected, and Cath-B activity was analyzed using Z-Arg Arg AMC as substrate. Data show mean (± SD) of at least four independent experiments carried out in duplicates. (C) Monocyte-derived macrophages in 12 well microtiter plates were incubated with or without 1µg/ml LPS for 3 h. Supernatant was collected, and Cath-B activity was analyzed using Z-Arg AMC as substrate. Data show mean (± SD) of at least four independent experiments carried out in duplicates. A representative experiment out of three is shown. Source data are provided as a Source Data file.

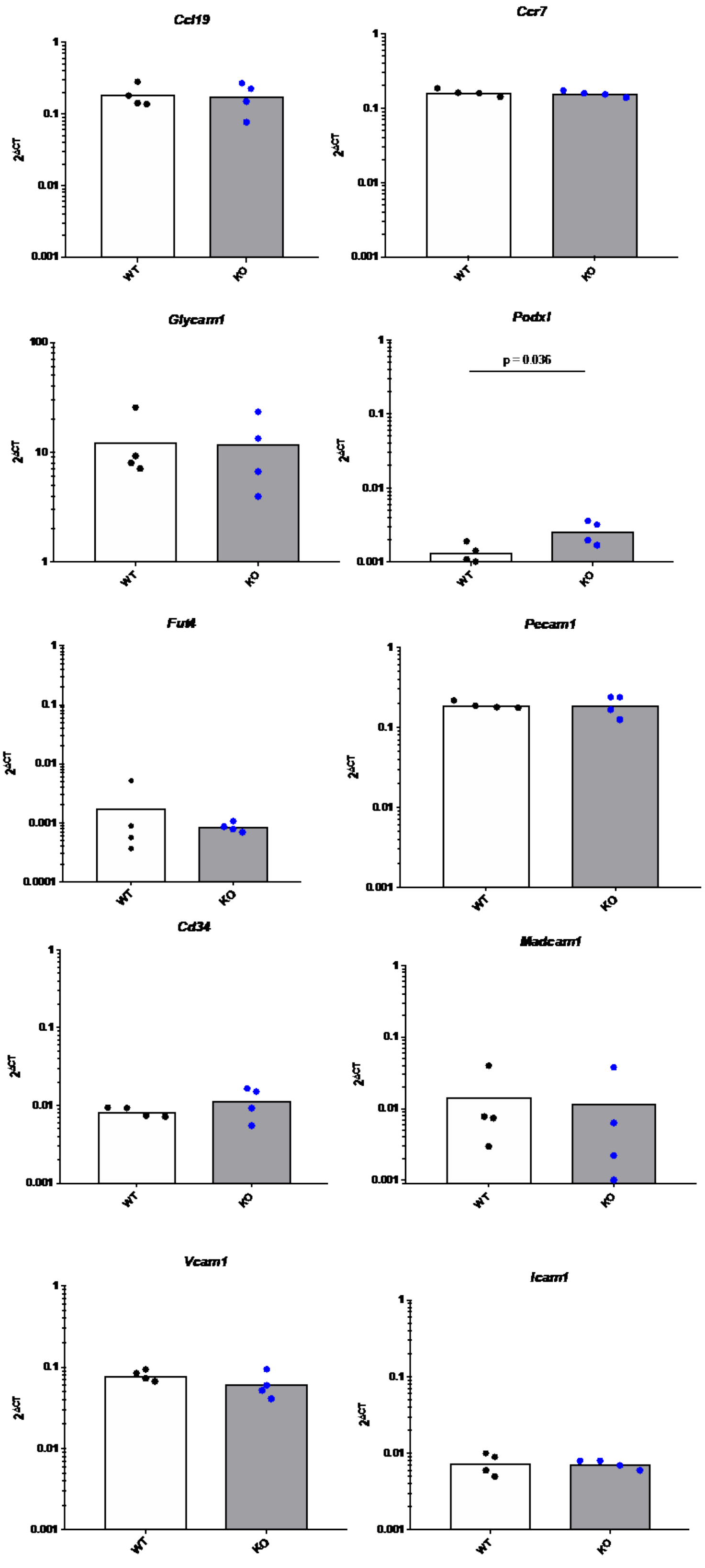

Supplementary Figure 8. RT-qPCR on whole lymph nodes: Analysis of a panel of genes relating to glycan synthesis and the formation of PNAd+ HEV scaffolds (Glycam1, Podxl, Cd34, Madcam1, FuctVII), cellular adhesion (Icam1, Vcam1, Pecam1) and chemokines (Ccl19, Ccr7). Significance was assessed using a Students T-test. Source data are provided as a Source Data file.

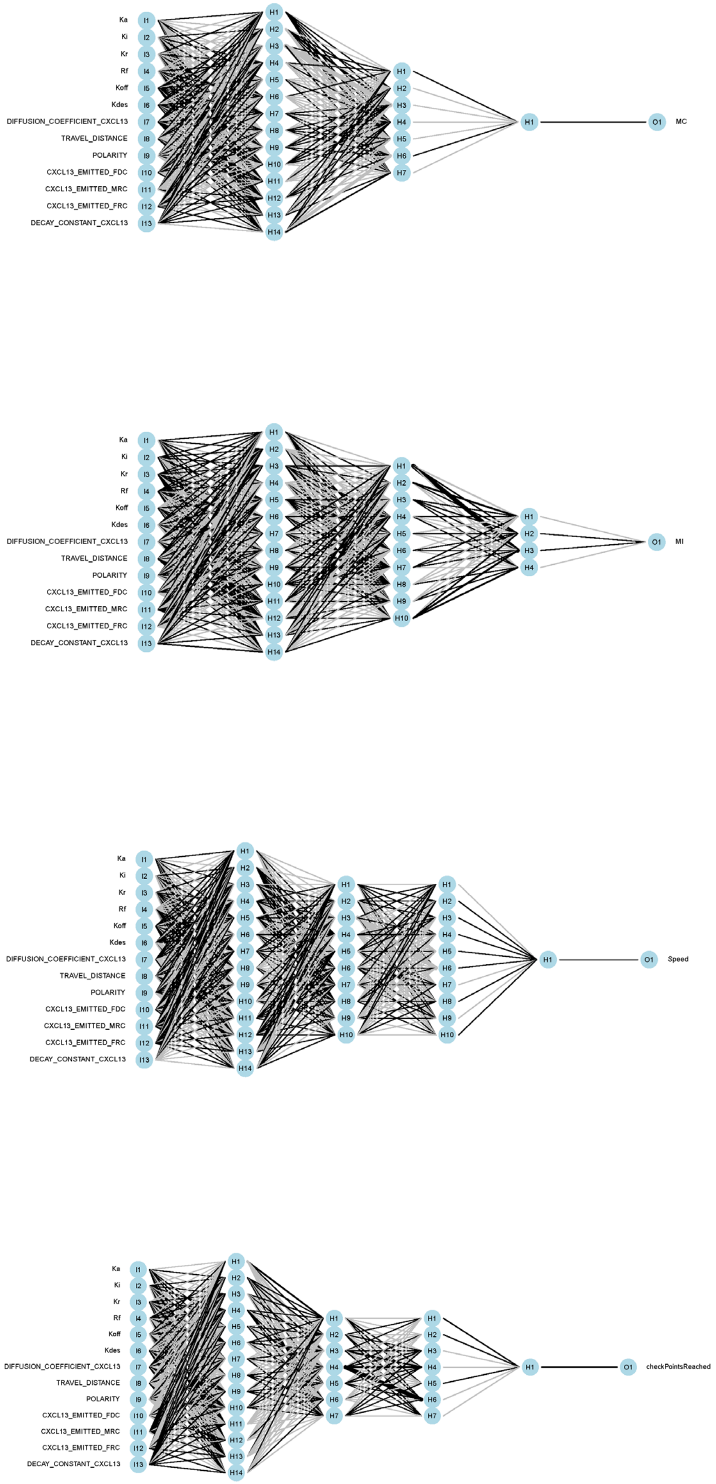

Supplementary Figure 9. Neural network structure for each output.

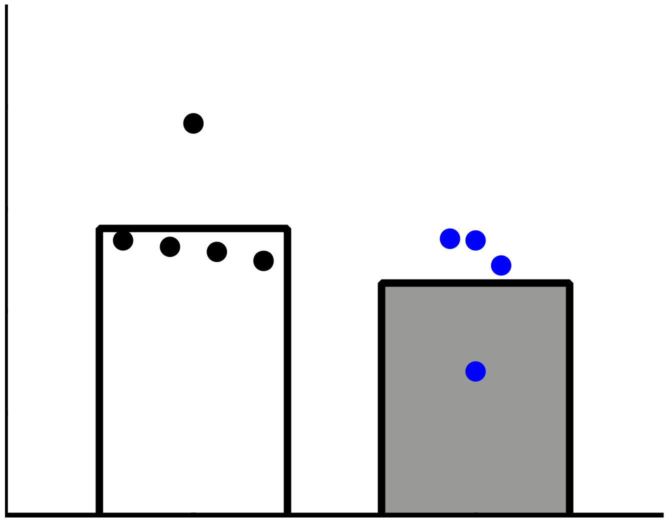

Supplementary Figure 10. Ratio of splenic entry of KO:WT B cells into either WT or Ctsb$^{-/-}$ recipients. To determine the relative efficiency of WT vs Ctsb$^{-/-}$ B cells to enter into WT or Ctsb$^{-/-}$ recipients, equal numbers of CSFE (ThermoFisher) labelled KO cells and CMTMR (ThermoFisher) labelled WT cells were transferred into corresponding recipient mice. The ratio of transferred B (B220^{+}) cells KO:WT was calculated by taking into account the relative efficiency of CFSE and CMTMR labelled survival post transfer by calculating the ratio of WT CSFE:WT CMTMR transferred cells. Source data are provided as a Source Data file.

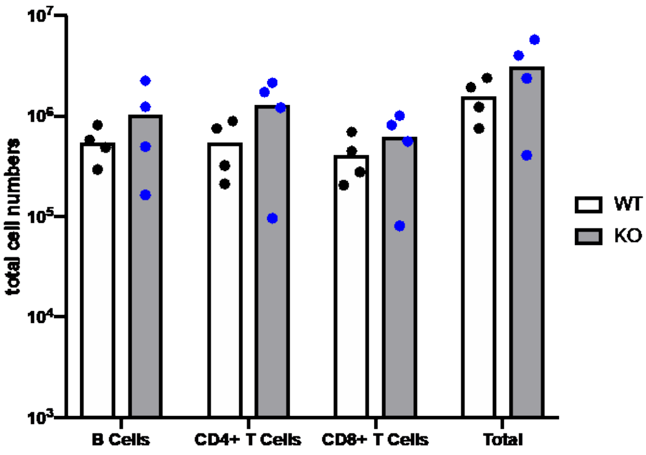

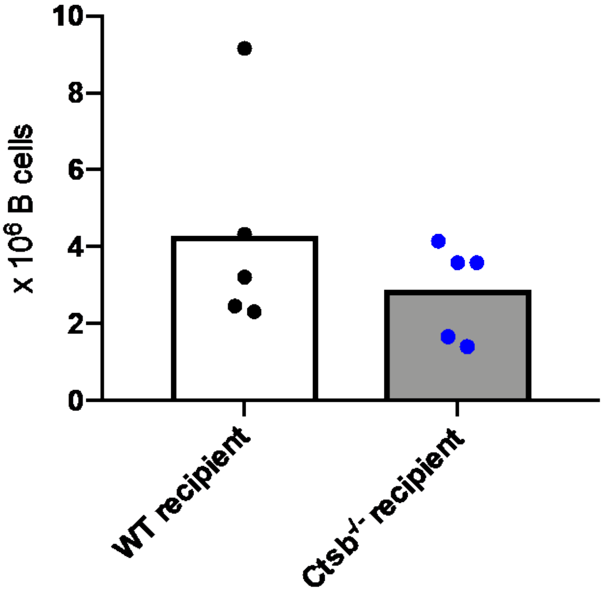

Supplementary Figure 11. Total cell numbers in WT and Ctsb$^{-/-}$ lymph nodes. (Top) absolute numbers of lymphocytes from LNs of WT and Ctsb$^{-/-}$ mice, data was taken from the experiment presented in Figure 6b (Bottom) The absolute number of B cells was determined by multiply the percentage of CD19^{+} B cells (measured by flow cytometry – FortessaX20) with absolute cell counts. Data was taken from the cell-transfer experiments described in Supplemental Figure 10. Source data are provided as a Source Data file.

Flow Cytometry Gating

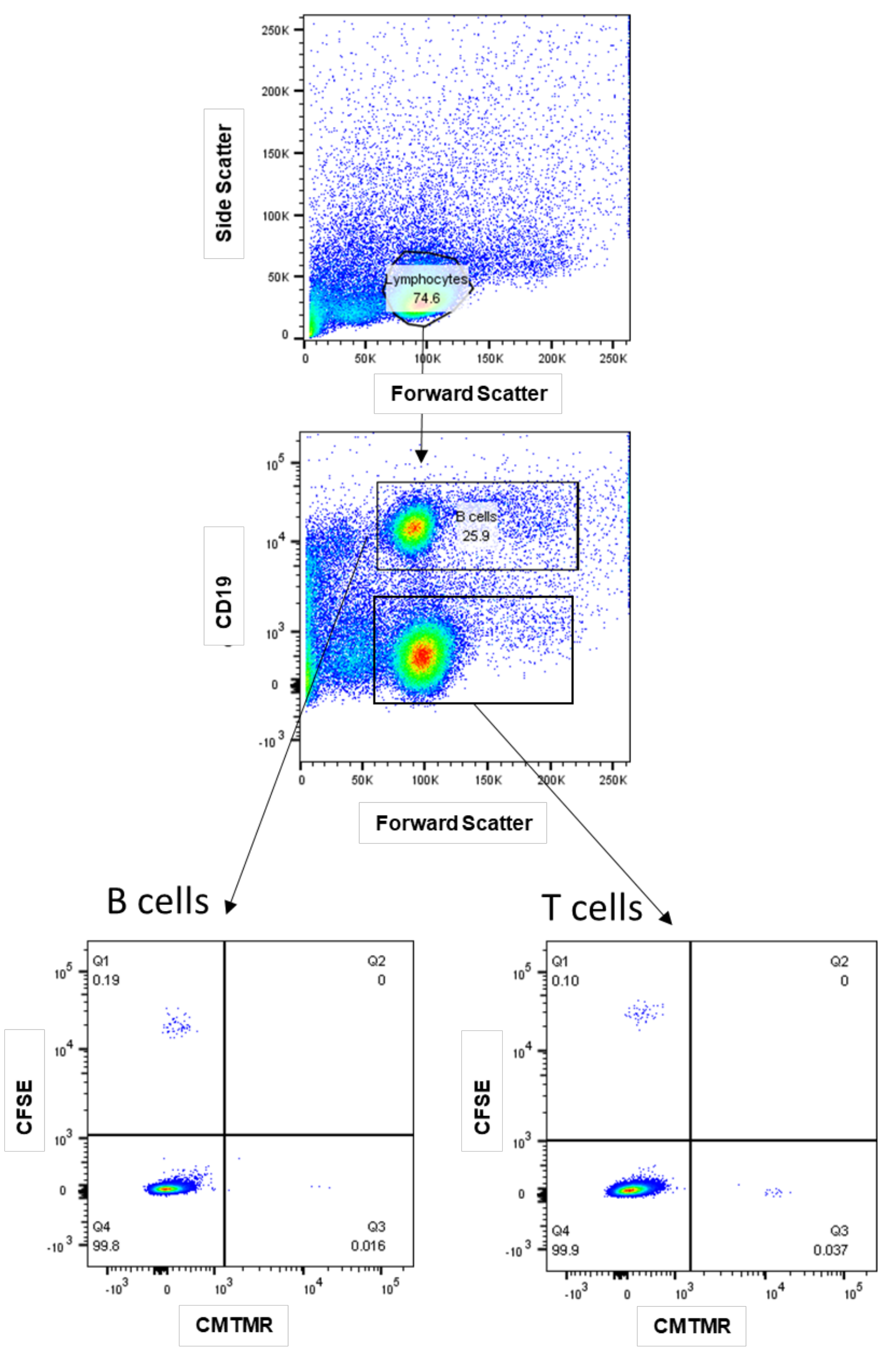

Supplementary Figure 12. Gating strategy to analyze cell entry rates into WT and Ctsb$^{-/-}$ lymph nodes. Cells were gated using forward and side scatter and then gated on CD19^{+} B cells and CD19^{-} lymphocytes (T cells). For each population off cells the relative frequency of transferred CFSE^{+} or CMTMR^{+} cells was calculated as described in the materials and methods section.

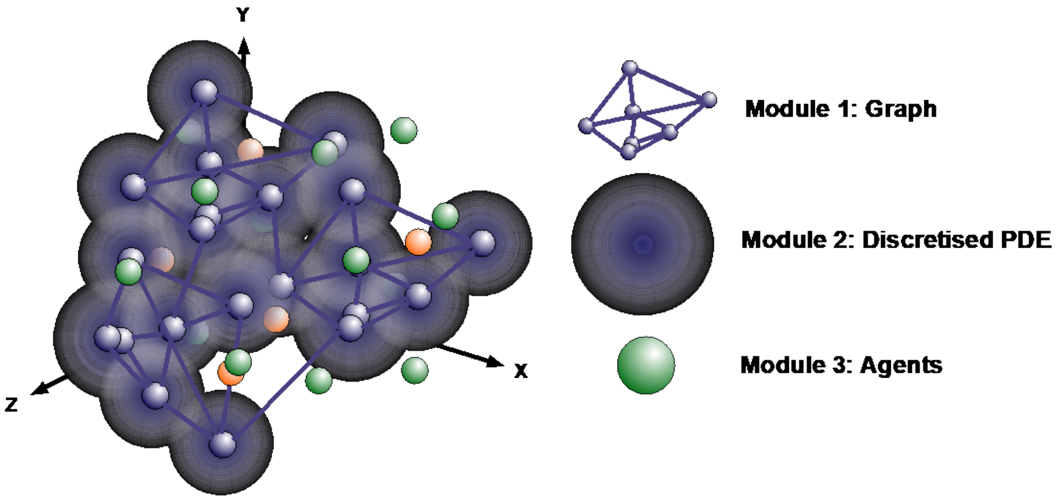

Supplementary Figure 13. Hybrid 3D multiscale representation of a follicle. In this system stromal cells are modelled as a graph (Module 1), chemokine diffusion is modelled as a discretised partial differential equation (Module 2), while B cells are modelled as rich agents which can interact with their local environment through a set of coupled differential equations and vector based calculations (Module 3).

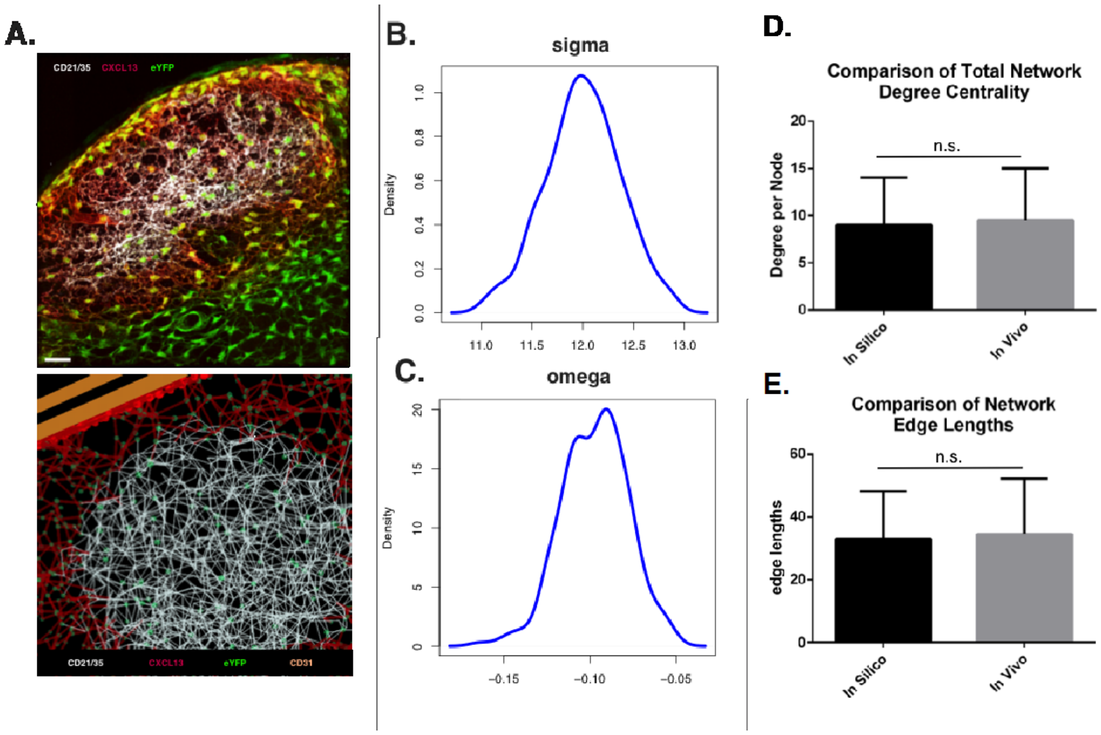

Supplementary Figure 14. Development and validation of the Stroma module. (a) Top figure shows a lymph node follicle showing tdTomato (red) and eYFP (green) expression from CXCL13-EYFP mice. FDCs are marked with an antibody against CD21/35 (white). Bottom figure shows in silico stromal networks. (b) This figure shows the distribution of sigma values obtained under baseline parameter values from 250 simulation runs. (c) This figure shows the distribution of omega values obtained under baseline parameter values from 250 simulation runs. Scale bar = 50µm. (d-e) No statistically significant differences were found between the median values for *in silico* and *in vivo* datasets as determined by a Mann-Whitney test with $p < 0.05$ representing a statistically significant result. Bars represent the median values and error bars represent the I.Q.R. Source data are provided as a Source Data file.

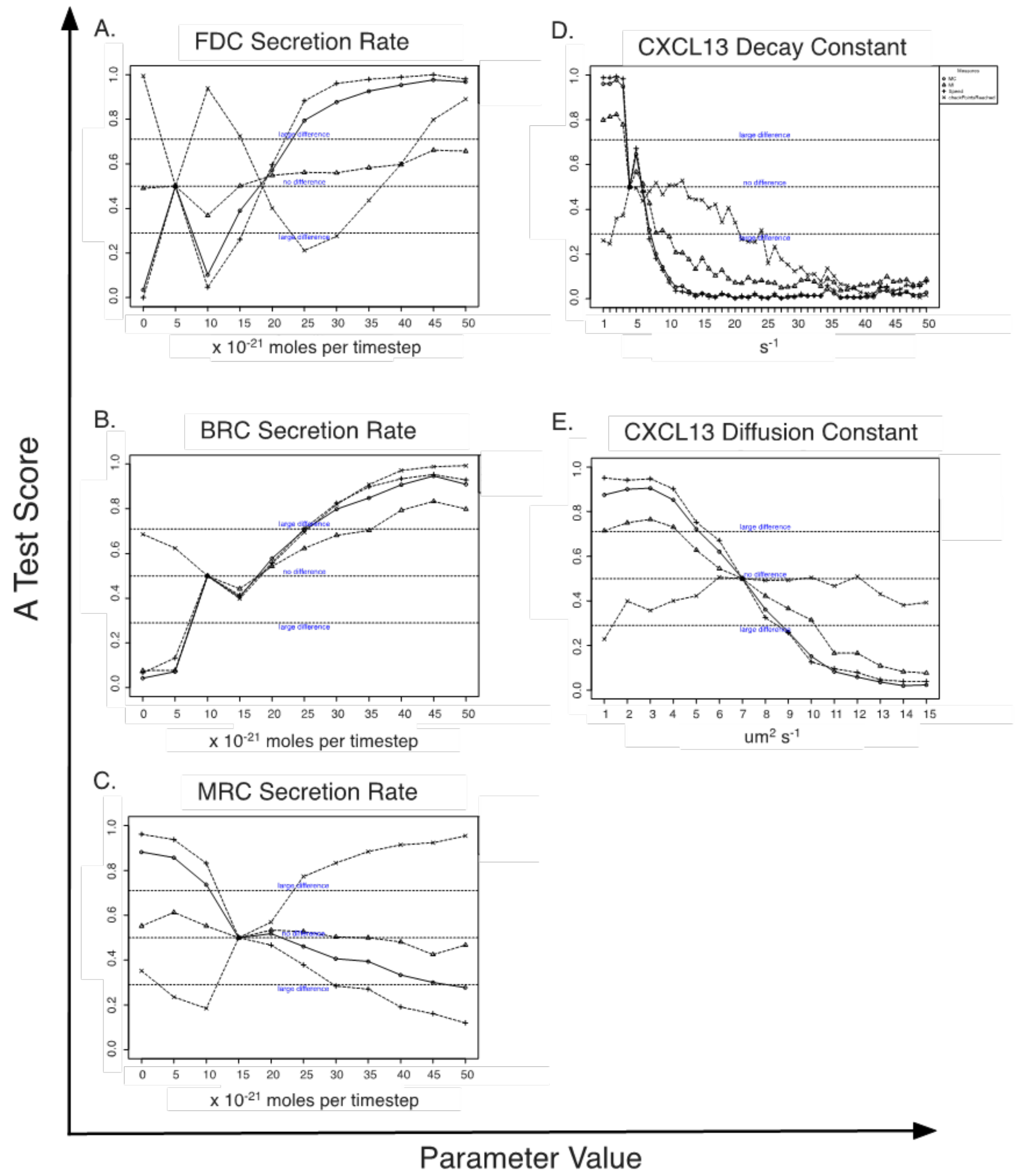

Supplementary Figure 15. A-test scores when OAT adjusting parameters which relate to CXCL13 bioavailability. Parameters *diffusion coefficient, cxcl13 emitted* and *decay constant* were incrementally changed within their likelihood distributions using an OAT parameter robustness approach. A significant alteration in simulation outputs from baseline behaviors was determined using the Vargha-Delaney A-Test. checkPointsReached ×; meandering index (MI) Δ; motility coefficient (MC) **o**; speed ✚. Source data are provided as a Source Data file

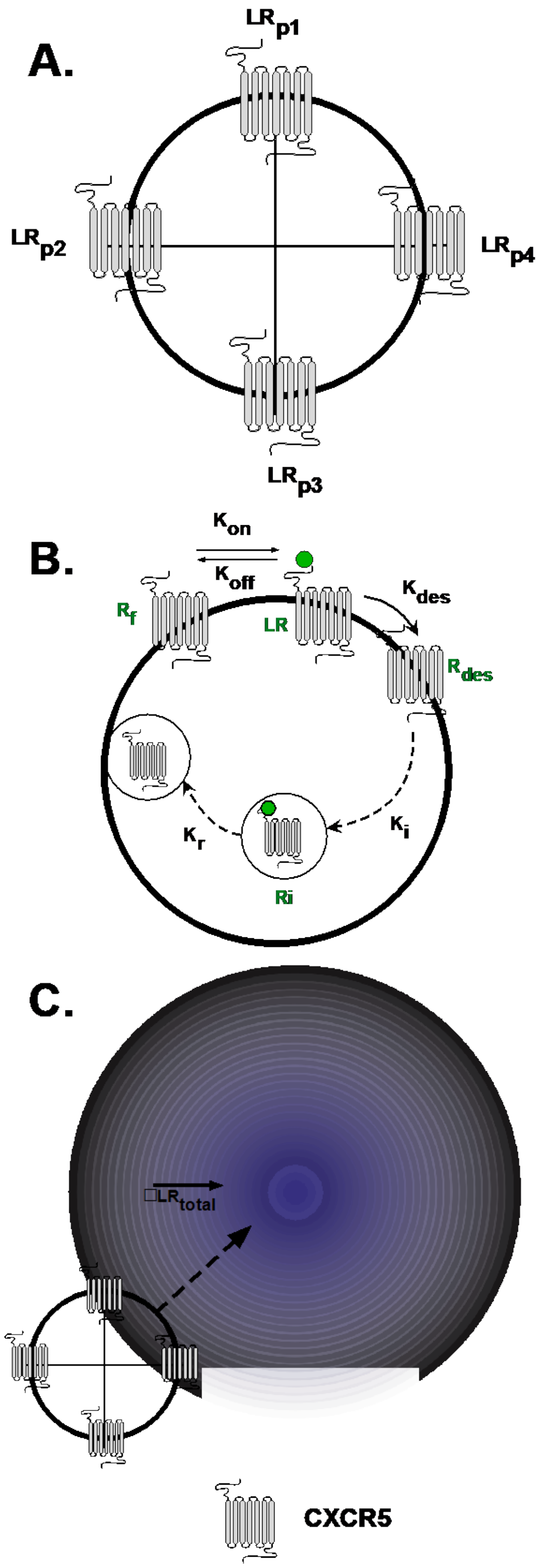

Supplementary Figure 16. Overview of receptor and migration kinetics in CXCL13Sim. (A) Each cell has 6 chemokine sampling pseudopods (4 are shown). (B) At each pseudopod, signalling is a function of local chemokine concentrations and receptor expression. Receptor expression is dynamic and subject to ligand association/dissociation as well as receptor desensitization, internalization and recycling. From the number of receptors signalling at each pseudopod, gradient vectors are calculated along 3 axes. (C) The overall net movement vector LRtotal determined by summing these vectors with a polarity vector representing cell persistence in a given direction.

Supplementary Table 1; Parameter Values to describe Topological Properties

Network	N	E	$\bar{L}$	$\bar{C}$	C_{glo}	σ	ω
B follicle	198 ± 39	1163 ± 242	4.17 ± 0.26	0.60 ± 0.02	0.57 ± 0.02	5.79 ± 1.05	-0.30 ± 0.01
ER	198 ± 39	1163 ± 242	2.41 ± 0.11	0.06 ± 0.01	0.06 ± 0.01	1.00 ± 0.004	0.91 ± 0.02
WS	198 ± 39	1189 ± 275	2.97 ± 0.23	0.51 ± 0.01	0.50 ± 0.01	6.99 ± 1.37	0.06 ± 0.04
Lattice	198 ± 39	1189 ± 275	8.84 ± 1.96	0.68 ± 0.01	0.68 ± 0.01	3.15 ± 0.24	-0.72 ± 0.05

Supplementary Table 1. Summary of parameters used to describe the topological properties of the B follicle and equivalent networks. N – number of nodes, E – number of edges, $\bar{L}$ – average shortest path length, $\bar{C}$ – average local clustering coefficient, C_{glo} – global clustering coefficient, σ – sigma factor, ω – omega factor, ER – Erdos-Renyi random network, WS – Watts-Strogatz small-world network, 2D ring lattice network. Data represent mean ± SD for n = 4 mice. ER and WS network parameters are summarized for 1000 simulations per mouse dataset. Source data are provided as a Source Data file.

Marker	Fluorophore	Supplier	Clone
**Anti-mouse CD19**	Alexa 488	eBioscience	eBio1D3
**Anti-Human CD19**	Alexa 488	Biolegend	HIB19
**Anti-Human CXCL13**	Unconjugated	R&D Biosystems	53610
**Anti-Human CD35**	Unconjugated	Santa Cruz	Ber-MAC-DRC
**Anti-Human CD68**	Unconjugated	Rabbit	EPR20545
**Anti-Human Heparan Sulfate**	Biotinylated	US Biological	10E4
**Anti-Human Cathepsin B**	Biotinylated	R&D Biosystems	Polyclonal
**Anti-Mouse CD3**	Alexa 488	Biolegend	17A2
**Anti-Mouse B220**	BV421/Alexa 488	Biolegend	RA3-6B2
**PNAd**	Alexa488	Nanotools	Meca79
**Podoplanin**	Alexa 594	Biolegend	8.1.1
**Anti-Mouse CD4**	Alexa 647	Biolegend	RM4-5
**Anti-Mouse CD19**	Alexa 647	Biolegend	6D5
**Anti-Mouse CD21/35**	Alexa 647	Biolegend	7E9

Supplementary Table 2: Antibodies used in the study

Parameter	Value	Unit	Range	Reference
**B Cell Size**	7	µm	Constant	[10]
**Total Number of B cells**	6000	cells	Constant	Measured
**Total Number of MRCs**	100	cells	Constant	Measured
**Total Number of FDCs**	~200	cells	Constant	Measured
**Total Number of BRCs**	~450	cells	Constant	Measured
**Proportion of Cognate Cells**	5	%	Constant	-
**Displacement constant**	7.4	µm min^{-1}	[1-10]	Calibrated
**Signal threshold**	10	ΔLR	Constant	[11,12]
**Maximum turn angle**	180	Degrees	Constant	[1]
**Total receptor number**	48,000	Receptors	[10,000-100,000]	[13]
**K_{on}**	4.8×10^5	M s^{-1}	[$1x10^5$-$1x10^6$]	[14]
**K_i**	0.0033	s^{-1}	[0.001-0.01]	[13,15]
**K_{des}**	0.075	s^{-1}	[0.01-0.1]	[13,15]
**K_r**	0.004	s^{-1}	[0.001-0.01]	[13,15]
**K_{off}**	0.0048	s^{-1}	[0.001-0.01]	[13,15]
**FDC secretion rate**	0.18	fg min^{-1} $cell^{-1}$	[0.1-0.5]	[16,17]
**RC secretion rate**	0.18	fg min^{-1} $cell^{-1}$	[0.1-0.5]	[16,17]
**CXCL13 decay rate**	0.007	s^{-1}	[0.0002-0.05]	[18,19]
**CXCL13 diffusion rate**	7.6	$µm^2$ s^{-1}	[0-146]	Measured (see section 3.3 for further details)
**Polarity**	0.475	-	0-1	Calibrated
**Random Polarity**	3.8	-	Constant	Calibrated

Supplementary Table 3: Summary of parameter values. For each parameter the name, baseline value and range used for uncertainty and sensitivity analyses is provided. Parameter values were determined experimentally or in cases where no direct experimental value exists, upper and lower limits were derived from indirect evidence, baseline values were then determined by fitting the model to experimental datasets (calibration). The model was further validated against migration data from $CXCR5^{-/-}$ B cells. The values for stromal cells are averaged over 250 runs with individual values varying to a small extent between runs due to stochastic network formation.

Supplementary Note 1: Metrics used in the topological characterization of follicular stromal cells

Degree centrality: The number of edges E connected to a given node N.

Edge length: The length of an edge in µm representing the minimal Euclidean distance between two nodes in 3D space, as opposed to the total physical length of interconnected cell protrusions.

Global clustering coefficient (C_{glo}): The global clustering coefficient is given by the ratio of the triangles and the connected triplet nodes in the network:

$$(1)\ \ C_{glo} = \frac{number\ of\ closed\ triplets}{number\ of\ connected\ triplets\ of\ nodes}$$

Average local clustering coefficient (C_{loc}): The local clustering coefficient c_i of a node *i* is defined as the number of edges e_i among neighbors of *i* divided by the total possible number of edges among its neighbors:

$$(2)\ \ c_i = \frac{2}{\delta_i(\delta_i - 1)} e_i\ ,\ \ 0 \le c_i \le 1$$

where δ_i represents the number of neighbors of node *i*.

The average local clustering coefficient $\bar{C}$ of a network with N nodes is the arithmetic mean of clustering coefficients of all the nodes *i*:

$$(3)\ \ \bar{C} = \frac{1}{N} \sum_{i=1;\, \delta_i>1}^{N} c_i\ ,\ \ 0 \le \bar{C} \le 1$$

Average shortest path length ($\bar{L}$): is defined as the arithmetic mean of all pairs of shortest distances between nodes *i* and *j*:

$$(4)\ \ \bar{L} = \frac{2}{N(N-1)} \sum_{i=1}^{N} \sum_{j=i+1}^{N} l_{ij}\ ,\ \ 1 \le \bar{L} < \infty$$

where l_{ij} is the length (number of edges) of the shortest path between nodes *i* and *j*, namely how many nodes one needs to pass in order to get from node *i* to node *j*. The maximum distance $\bar{L}_{max}$ is called the diameter of the network. In case of a complete

network where all possible connections are present, all the node distances $l_{ij} = 1$, thus the sum $\sum_{i=1}^{n}\sum_{j=i+1}^{n} l_{ij} = \frac{N(N-1)}{2}$, which gives the minimal $\bar{L}_{min} = 1$.

Sigma factor (*σ*) : The small-world measure σ is defined as:

$$(5) \qquad \sigma = \frac{\bar{C}/C_R}{\bar{L}/L_R}, \;\; 1 \leq \sigma < \infty$$

where C_R and L_R are the average clustering coefficient and average shortest path length of an equivalent random network averaged across 1000 simulation runs. The equivalent random network has the same number of nodes and edges as the target network and is generated using the Erdos-Renyi model. A network has small-world properties if $\bar{C} \gg C_R, \bar{L} \geq L_R$ and therefore $\sigma > 1$.

Omega factor (*ω*): The small-world measure ω is defined as:

$$(6) \quad \omega = \frac{L_R}{\bar{L}} - \frac{\bar{C}}{C_L}, \;\; -1 < \omega < 1$$

where C_L is the average local clustering coefficient of an equivalent lattice network and L_R is the average shortest path length of an equivalent Erdos-Renyi random network (1000 simulation runs). A network will be classified as a small-world network if: $-0.5 \leq \omega \leq 0.5$.

Supplementary Note 2: Computational Resources

An individual simulation run takes approximately 25 minutes under baseline parameter values on an Apple MacBook Pro, 2.5GHz Intel four core i7, 16GB RAM. A 500-sample LHC experiment requires 100,000 executions (due to the stochastic outputs of CXCL13Sim, multiple runs are required to evaluate a single parameter set as detailed further below) with simulation runs performed on the York Advanced Research Computing Cluster, a resource of 70 nodes, 138 processors, 1462 cores, and 10.2TB RAM. This analysis can also be performed using the emulator on the Apple MacBook Pro detailed above on the order of seconds.

Supplementary Note 3: Overview of the Multiscale Simulator

This section provides a brief overview of the simulator platform. A full description of model simulator design, development and validation, as well as associated source code, is available from:

https://www.york.ac.uk/computational-immunology/software/cxcl13sim.

CXCL13Sim is a 3D hybrid multiscale model developed using the CoSMoS (Complex System Modelling and Simulation) process, a framework to guide the modelling and analysis of complex systems [2]. The model has a modular architecture with reticular cells cells, B cells and chemokines representing a distinct functional subunit (Supplemental Figure 13).

In this system *in silico* reticular cells are modeled as a graph of nodes and edges (Module 1). Reticular cells secrete chemokine, which is represented by a double precision floating point number on a discretized grid with diffusion modeled using a discretized partial differential equation (Module 2) [3]. B cells are modeled as agents that adjust their behaviors with respect to vector and ordinary differential equation-based calculations adapted from a published scheme which explicitly accounts for gradient detection and the dynamics of GPCR expression on the cell surface (Module 3) [4,5]. These modules are detailed further in the following sections.

The simulation platform was implemented using Java and the MASON agent-based modelling library version 19 [6], (http://cs.gmu.edu/~eclab/projects/mason) in an iterative process of implementation, validation and refactoring using Acceptance Test-Driven Development (ATDD)[7]. Tests are continually assessed and refined as the project progresses and are incorporated into an automated regression framework using the java library JUnit (available from http://junit.org/junit4/) to ensure that new code does not disrupt existing functionality, expediting the development process.

To calibrate simulator behaviours to experimentally observed migration patterns (Supplementary Figure 3), the 13 free model parameters (Supplementary table 3) were systematically changed and outputs were compared to *in vivo* multiphoton datasets. Fluorescently labelled wild-type and $CXCR5^{-/-}$ B cells were adoptively transferred into WT hosts and their distribution and migration patterns inside popliteal lymph nodes analysed using selective plane illumination microscopy, which preserves the three-

dimensional organ structure. The median values for 250 simulation runs are 1.15 for the meandering index, 13.28 for motility coefficient and 8.11 for speed. Comparison of a single *in silico* run with *in vivo* datasets showed no significant differences between motility coefficients, meandering indices and velocities for (a) wild-type or (b) $CXCR5^{-/-}$ B cells; assessed using a Mann-Whitney test at a significance level of 5%. The additional 9 parameters were fixed at empirically determined or calibrated values representing key attributes such as cell size and the density of the stromal cell network that are not designed to change between simulation runs.

To facilitate the interpretation of simulator-derived results we quantify parametric (using local and global approaches) and aleatory uncertainty using the R software package SPARTAN[8,9].

4.1 Module 1: Stromal

In silico stromal networks are generated using an adaptation of the algorithm developed by Kislitsyn *et al.* (2015). The algorithm stochastically builds a network from an initial node, picking the nearest non-expanded node and generates a set of vectors that will eventually become edges to new nodes. Each vector has a direction and length randomly chosen, but conforming to values derived from experimental data, and the directions are chosen such that the sum of all the vectors in the set is approximately zero. A new node is then created at the end of each vector, and an edge connects them. This process is repeated to generate a graph. Branches between edges are added by creating a vector connecting the midpoints of each edge, subject to subtype-specific constraints on the maximum edge length and the local density such that the degree centrality matches our *in vivo* datasets. This approach yields networks with median sigma and omega values of **12.00** and **-0.097** respectively, confirming that the network has small world properties (Supplementary Figure 14). The discrepancy between *in vivo* and *in silico* sigma values was expected as sigma scales with network size and the *in silico* follicle, is approximately 4 times the volume of the tissue section used to perform the topological mapping. Comparison of the median values for both edge lengths and degree centralities for *in silico* and *in vivo* networks shows no statistically significant differences (Supplementary Figure 14).

4.2. Module 2: Chemokine

The scheme we implement is a discretized form of the heat equation[3]. This mathematical construct is capable of isotropic diffusion[3], can diffuse to an arbitrary number of neighbors and is applicable to linear, planar, spatial and n-dimensional implementations. In this scheme, chemokine molecules diffuses through a discrete 3D environment where the number of moles of chemokine molecules in each grid space *(x,y,z)* is denoted $\varphi(x,y,z)$. The change in the spatial distribution of molecules is then subject to the following simultaneously occurring processes *(i) production (ii) diffusion (iii) decay and (iv) consumption*. As we are using agents to model individual cells, terms (i) and (iv) emerge from the simulation. Chemokine is secreted by each stromal cell at a fixed rate and is removed from the grid at a rate that is proportional to its current value λ.

$$(1)\ \varphi(x,y,z)_{t+1} = (1-\lambda)\varphi(x,y,z)_t$$

where *t* represents the time step. At each discrete time step chemokine diffuses to the grid spaces adjacent and diagonally adjacent to each grid space. The coefficient for the amount of chemokine diffused to each grid space is:

$$(2)\ A\big(\varphi(x)-\varphi(y)\big)e^{\frac{-d_y^2}{\mu}}$$

$$(3)\ \mu = 4Dt$$

where chemokine in grid space *x*, $\varphi(x)$, is being diffused to grid space *y*, d_y^2 is the distance squared between *x* and *y*, *D* is the diffusion constant, *t* is the time step, and A is a normalizing constant that ensures the total amount being diffused is less than or equal to the amount that exists:

$$(4) A\sum_{y=1}^{n} e^{\frac{-d_y^2}{\mu}} = 1$$

for an arbitrary grid space where n represents the number of surrounding grid spaces in the diffusion neighborhood, which in CXCL13Sim is a 3D Moore neighborhood with 26 neighboring grid spaces. The borders of the grid follow Dirichlet boundary conditions. When modeling diffusion in discrete space, the speed at which chemokine

diffuses is limited by the time step and size of the diffusion neighborhood. To account for this, the time step we use is $\frac{\delta^2}{kD}$ where δ is the length of an edge of the discretized grid spaces, D is the diffusion coefficient, and k is a constant, empirically determined in order to match the measured diffusion speed with the mathematically derived value for mean-squared displacement for a given diffusion constant. The diffusion grid is updated every second while agents are updated once every minute and are assumed quasi-static with respect to diffusion. The baseline value of the diffusion constant was informed using preliminary measures of CXCL13 mobility in follicles of lymph node tissue sections obtained using the single-molecule super-resolution imaging approach described in Miller and Cosgrove (2018). To assess whether the discrepancy between our baseline value of 7.6 microns squared per second and the final published value of 6.6 microns squared per second would affect emergent *in silico* cellular behaviors we performed a local sensitivity analysis (Supplementary Figure 15). Results from this analysis show that changing the diffusion constant value between 6-8 microns squared per second did not lead to a statistically significant alteration in cellular behaviors *in silico* and consequently 7.6 was used as the baseline value for the diffusion constant. In addition, we note that the cxcl13 decay constant (Supplementary Figure 15) is a highly influential parameter with small increases in its value significantly changing agent behaviours. This result is corroborated by our MOEA analysis in Figure 2 of the manuscript which shows that our calibrated value is to the extreme left of the distribution of our Pareto optimal solutions. This may introduce bias and thus we exercise caution when assessing the influence of this parameter on the biological system.

4.3. Module 3: Lymphocytes

To account for dense lymph node environment lymphocyte migration must take into account interactions with other cell types. As time proceeds in fixed discrete intervals we treat both the movement vector of lymphocytes and the edge of the stromal cell as lines. To determine if the two agents are interacting we calculate whether the closest point between the two lines is less than the sum of their diameters. Additionally, cells may interact with each other, however the cell structure is dynamic, and cells are observed to slide over one another. To account for this, once an agent has determined the direction in which to move, the probability that the cell can move towards the target

location is determined as $e^{-\delta}$, where δ is the absolute number of cells in the target location.

Chemotaxis, and chemokine receptor internalization and recycling are key mechanisms governing the fine-tuning of responses to chemokines *in vivo* [4,20,21]. To model these phenomena *in silico,* we adapt the scheme developed by Lin *et al* [4,5]. In this scheme, an agent, samples local chemokine concentrations using 6 sampling pseudopodia (Supplementary Figure 16). At each pseudopod there are a population of receptors on the cell surface [R_f] that are free to bind ligand [L] at a rate K_{on}. Receptor dynamics are controlled by a set of ordinary differential equations solved on a per agent basis using a 4th order Runge-Kutta scheme [22]. Ligation of the chemokine to its respective receptor [LR] leads to downstream signaling cascades and localization of actin with ligand dissociating at a rate K_{off}. Following binding receptors are desensitized at a rate K_{des}, internalized at a rate K_i and are recycled at a rate K_r (Supplementary Figure 16). From calculating [LR] at each pseudopod, a gradient vector, $\overrightarrow{LR}$ is calculated across the cell along 3 axes with ρ representing each individual pseudopod. If $\overrightarrow{LR}$ exceeds a threshold then the cell will become chemotactic, with the overall orientation vector of the cell $\overrightarrow{LR_{total}}$ taken as a sum of $\overrightarrow{LR}$ with a leading edge vector $\overrightarrow{LR_m}$ that accounts for the orientation of the cell from the previous time step.

$$(5)\ \overrightarrow{LR} = \left([LR_{\rho 1}] - [LR_{\rho 4}]\right) + \left([LR_{\rho 2}] - [LR_{\rho 5}]\right) + \left([LR_{\rho 3}] - [LR_{\rho 6}]\right)$$

$$(6)\ \overrightarrow{LR_{total}} = \frac{\alpha\overrightarrow{LR} + \overrightarrow{LR_m}}{\left|\alpha\overrightarrow{LR} + \overrightarrow{LR_m}\right|}$$

$$(7)\ \frac{d[\mathrm{LR}]}{dT} = K_{on}[L][R_f] - K_{des}[LR] - K_{off}[LR]$$

$$(8)\ \frac{d[R_f]}{dT} = K_r[R_i] - K_{on}[L][R_f] + K_{off}[LR]$$

$$(9)\quad \frac{d[R_i]}{dT} = K_i[R_{des}] - K_r[R_i]$$

$$(10) \quad \frac{d[R_{des}]}{dT} = K_{des}[LR] - K_i[R_{des}]$$

In the presence of chemotactic gradients actin flows polarize at the leading edge of the cell thus the relative weighting between $\overrightarrow{LR}$and $\overrightarrow{LR_m}$ is scaled by a constant α to represent the persistence of the cell; the value of α is dependent on the chemotactic state of the agent. As a universal coupling exists between actin flows and cell speed [23] and relate the increase in velocity $v*$ observed during chemokinesis to cell persistence α using the following expression:

$$(11) \quad v* = \frac{LN(\alpha)}{\gamma}$$

$$\alpha = \begin{cases} \alpha_1, & if\ chemotactic \\ \alpha_2, & if\ not\ chemotactic \end{cases}$$

where LN is the natural log function. The value of the scalar term γ was determined empirically, by fitting the model to WT migration data and verifying against CXCR5$^{-/-}$ migration patterns [1]. A number of automated tests, were developed to ensure that total receptor values are conserved over time, and that agents move towards high concentrations of chemokines when expressing CXCR5.

Supplemental References:

1. Coelho, F. M. *et al.* Naive B-cell trafficking is shaped by local chemokine availability and LFA-1–independent stromal interactions. *Blood* **121**, 4101–4109 (2013).
2. Alden, K., Timmis, J., Andrews, P. S., Veiga-Fernandes, H. & Coles, M. C. Pairing experimentation and computational modeling to understand the role of tissue inducer cells in the development of lymphoid organs. *Inflammation* **3**, 172 (2012).
3. Grajdeanu, A.. MODELING DIFFUSION IN A DISCRETE ENVIRONMENT.
4. Lin, F. & Butcher, E. C. Modeling the Role of Homologous Receptor Desensitization in Cell Gradient Sensing. *J. Immunol. Baltim. Md 1950* **181**, 8335–8343 (2008).
5. Wu, D. & Lin, F. Modeling Cell Gradient Sensing and Migration in Competing Chemoattractant Fields. *PLoS ONE* **6**, e18805 (2011).
6. Luke, S., Cioffi-Revilla, C., Panait, L., Sullivan, K. & Balan, G. MASON: A Multiagent Simulation Environment. *SIMULATION* **81**, 517–527 (2005).
7. Sommerville, I. *Software Engineering*. (Pearson, 2010).
8. Vargha, A. & Delaney, H. D. A Critique and Improvement of the 'CL' Common Language Effect Size Statistics of McGraw and Wong. *J. Educ. Behav. Stat.* **25**, 101–132 (2000).
9. Alden, K. *et al.* Spartan: a comprehensive tool for understanding uncertainty in simulations of biological systems. *PLoS Comput. Biol.* **9**, e1002916 (2013).
10. Monroe, J. G. & Cambier, J. C. Sorting of B lymphoblasts based upon cell diameter provides cell populations enriched in different stages of cell cycle. *J. Immunol. Methods* **63**, 45–56 (1983).
11. Zigmond, S. H. Consequences of chemotactic peptide receptor modulation for leukocyte orientation. *J. Cell Biol.* **88**, 644–647 (1981).
12. Herzmark, P. *et al.* Bound attractant at the leading vs. the trailing edge determines chemotactic prowess. *Proc. Natl. Acad. Sci. U. S. A.* **104**, 13349–13354 (2007).
13. Sh, Z., Sj, S. & Da, L. Kinetic analysis of chemotactic peptide receptor modulation., Kinetic analysis of chemotactic peptide receptor modulation. *J. Cell Biol. J. Cell Biol.* **92, 92**, 34, 34–43 (1982).
14. Barroso, R. *et al.* EBI2 regulates CXCL13-mediated responses by heterodimerization with CXCR5. *FASEB J. Off. Publ. Fed. Am. Soc. Exp. Biol.* **26**, 4841–4854 (2012).
15. Tilo Beyer, M. M.-H. Modeling emergent tissue organization involving high-speed migrating cells in a flow equilibrium. *Phys. Rev. E Stat. Nonlin. Soft Matter Phys.* **76**, 021929 (2007).

16. Luther, S. A. *et al.* Differing Activities of Homeostatic Chemokines CCL19, CCL21, and CXCL12 in Lymphocyte and Dendritic Cell Recruitment and Lymphoid Neogenesis. *J. Immunol.* **169**, 424–433 (2002).
17. Gunn, M. D. *et al.* A B-cell-homing chemokine made in lymphoid follicles activates Burkitt's lymphoma receptor-1. *Nature* **391**, 799–803 (1998).
18. Wang, Y. & Irvine, D. J. Convolution of chemoattractant secretion rate, source density, and receptor desensitization direct diverse migration patterns in leukocytes. *Integr. Biol. Quant. Biosci. Nano Macro* **5**, 481–494 (2013).
19. Phair, R. D. & Misteli, T. Kinetic modelling approaches to in vivo imaging. *Nat. Rev. Mol. Cell Biol.* **2**, 898–907 (2001).
20. Bennett, L. D., Fox, J. M. & Signoret, N. Mechanisms regulating chemokine receptor activity. *Immunology* **134**, 246–256 (2011).
21. Rot, A. & von Andrian, U. H. Chemokines in innate and adaptive host defense: basic chemokinese grammar for immune cells. *Annu. Rev. Immunol.* **22**, 891–928 (2004).
22. Press, W. H., Teukolsky, S. A., Vetterling, W. T. & Flannery, B. P. *Numerical Recipes 3rd Edition: The Art of Scientific Computing*. (Cambridge University Press, 2007).
23. Maiuri, P. *et al.* Actin Flows Mediate a Universal Coupling between Cell Speed and Cell Persistence. *Cell* **161**, 374–386 (2015).
