# Supplementary material for: B cell zone reticular cell microenvironments shape CXCL13 gradient formation
Source: Nat Commun. 2020 Jul 22;11:3677. doi: 10.1038/s41467-020-17135-2 (PMC7376062; doi:10.1038/s41467-020-17135-2)
Supplement: Supplementary file 3 — Reporting Summary [file 41467_2020_17135_MOESM3_ESM.pdf]

nature research

Corresponding author(s): Mark Coles

Last updated by author(s): Mar 3, 2020

Reporting Summary

Nature Research wishes to improve the reproducibility of the work that we publish. This form provides structure for consistency and transparency in reporting. For further information on Nature Research policies, see Authors & Referees and the Editorial Policy Checklist.

Statistics

For all statistical analyses, confirm that the following items are present in the figure legend, table legend, main text, or Methods section.

n/a	Confirmed	
☐	☒	The exact sample size (*n*) for each experimental group/condition, given as a discrete number and unit of measurement
☐	☒	A statement on whether measurements were taken from distinct samples or whether the same sample was measured repeatedly
☐	☒	The statistical test(s) used AND whether they are one- or two-sided<br>*Only common tests should be described solely by name; describe more complex techniques in the Methods section.*
☐	☒	A description of all covariates tested
☐	☒	A description of any assumptions or corrections, such as tests of normality and adjustment for multiple comparisons
☐	☒	A full description of the statistical parameters including central tendency (e.g. means) or other basic estimates (e.g. regression coefficient) AND variation (e.g. standard deviation) or associated estimates of uncertainty (e.g. confidence intervals)
☐	☒	For null hypothesis testing, the test statistic (e.g. F, t, r) with confidence intervals, effect sizes, degrees of freedom and P value noted<br>*Give P values as exact values whenever suitable.*
☒	☐	For Bayesian analysis, information on the choice of priors and Markov chain Monte Carlo settings
☐	☒	For hierarchical and complex designs, identification of the appropriate level for tests and full reporting of outcomes
☐	☒	Estimates of effect sizes (e.g. Cohen's *d*, Pearson's *r*), indicating how they were calculated

Our web collection on statistics for biologists contains articles on many of the points above.

Software and code

Policy information about availability of computer code

Data collection	Software code is freely available in a Github. Transparent evidence base for the model is documented on website and can be downloaded using the link in the manuscript
Data analysis	All analytical techniques are described including the mathematical equations for data analysis, software for image analysis was IMARIS, ZEN and FUJI ImageJ. Flow cytometry data was analysed using FLOWJO.

For manuscripts utilizing custom algorithms or software that are central to the research but not yet described in published literature, software must be made available to editors/reviewers. We strongly encourage code deposition in a community repository (e.g. GitHub). See the Nature Research guidelines for submitting code & software for further information.

Data

Policy information about availability of data

All manuscripts must include a data availability statement. This statement should provide the following information, where applicable:
- Accession codes, unique identifiers, or web links for publicly available datasets
- A list of figures that have associated raw data
- A description of any restrictions on data availability

Source Data is provided in Zip folder. All Raw datasets (1.5TB zip file) that support the findings of this study are available from the corresponding author upon reasonable request.

Field-specific reporting

Please select the one below that is the best fit for your research. If you are not sure, read the appropriate sections before making your selection.

☒ Life sciences ☐ Behavioural & social sciences ☐ Ecological, evolutionary & environmental sciences

For a reference copy of the document with all sections, see nature.com/documents/nr-reporting-summary-flat.pdf

Life sciences study design

All studies must disclose on these points even when the disclosure is negative.

Sample size	Sample sizes were determined using power analysis to determine minimum sample sizes required. All experiments were independently repeated to reduce effects.
Data exclusions	No data was excluded from the analysis. All raw data is available upon request as a ZIP Folder (1.5TB), source data is provided in ZIP folder on the Nature Communications website. Additional data and information is provided on the resource website listed in the manuscript
Replication	Mouse experiments were performed on two different labs to validate the results, data analysis was blinded and all experiments contained sufficient animals to provide statistical power for experimental results.
Randomization	Were appropriate samples were randomized to permit blinding of image analysis
Blinding	Analysis of imaging data was blinded to prevent bias.

Reporting for specific materials, systems and methods

We require information from authors about some types of materials, experimental systems and methods used in many studies. Here, indicate whether each material, system or method listed is relevant to your study. If you are not sure if a list item applies to your research, read the appropriate section before selecting a response.

Materials & experimental systems

n/a	Involved in the study	
☐	☒	Antibodies
☒	☐	Eukaryotic cell lines
☒	☐	Palaeontology
☐	☒	Animals and other organisms
☐	☒	Human research participants
☒	☐	Clinical data

Methods

n/a	Involved in the study	
☒	☐	ChIP-seq
☐	☒	Flow cytometry
☒	☐	MRI-based neuroimaging

Antibodies

Antibodies used	MECA-79 Alexa488 (Nanotools (Custom Product), 1 in 200 dilution); PDPN Alexa 594 (Biolegend (8.1.1)(Cat. 127414); B220 Alexa488 (Biolegend (RA-6B2)(Cat. 103225), 1 in 200 dilution); CD4 Alexa647 (Biolegend (RM4-5)(Cat. 100516), 1 in 200 dilution); CD21/35 Alexa647 (Biolegend (7E9)(Cat. 123424) 1 in 200 dilution); and CD19 Alexa 647 (Biolegend (6D5)(Cat. 11512), 1 in 200 dilution).
Validation	All antibodies used were titered for optimal staining, all antibodies were extensively used in experiments and well understood clones thus there was high confidence in the clones

Animals and other organisms

Policy information about studies involving animals; ARRIVE guidelines recommended for reporting animal research

Laboratory animals	C57BL6 wild type, litter mate controls and Cathepsin B deficient mice were bred in SPF facility. All experiments were performed in compliance with the ARRIVE guidelines
Wild animals	N/A
Field-collected samples	N/A
Ethics oversight	Ethics were oversean by local AWERB committee in York (UK), Oxford (UK) and St. Gallen (Switzerland) full approval numbers and information is provided in the manuscript

Note that full information on the approval of the study protocol must also be provided in the manuscript.

Human research participants

Policy information about studies involving human research participants

Population characteristics	All human tonsil and lymph node samples were obtained from healthy volunteers aged from 18 - 40 with an average age of 26. Only samples from non-smokers were collected undergoing elective surgery for tonsils or were from healthy liver transplants (human liver)
Recruitment	Recruitment was performed using double consent prior to tonsil donation, lymph nodes were obtained through consent for multi-organ donation as approved by Medical Ethics Committee of the Erasmus MC.
Ethics oversight	Tonsils were collected under NRES REC 12/NE/0360 approved study (IRAS: 114771) to MCC. Hepatic lymph nodes were collected during multi-organ donation procedures, after approval by the Medical Ethical committee of the Erasmus MC (MEC-2014-060) by WGP.

Note that full information on the approval of the study protocol must also be provided in the manuscript.

Flow Cytometry

Plots

Confirm that:

☒ The axis labels state the marker and fluorochrome used (e.g. CD4-FITC).

☒ The axis scales are clearly visible. Include numbers along axes only for bottom left plot of group (a 'group' is an analysis of identical markers).

☒ All plots are contour plots with outliers or pseudocolor plots.

☒ A numerical value for number of cells or percentage (with statistics) is provided.

Methodology

Sample preparation	All samples were processed
Instrument	BD Fortessa X-20
Software	BD FACSDIVA for data aquisition, FLOWJO (Becton Dickinson) was used for data analysis
Cell population abundance	No cell sorting was performed in the studies
Gating strategy	The gating strategy is described in supplemental figure 12 and described in the methods section

☒ Tick this box to confirm that a figure exemplifying the gating strategy is provided in the Supplementary Information.
