# Supplementary material for: B cell zone reticular cell microenvironments shape CXCL13 gradient formation
Source: Nat Commun. 2020 Jul 22;11:3677. doi: 10.1038/s41467-020-17135-2 (PMC7376062; doi:10.1038/s41467-020-17135-2)

Time course CXCL13 digest_1
Wed Dec 11 16:07:08 CET 2019
Project: 040708 Prot Gel CXCL13 digest timecourse
Scan: newCXCL13 time course digest

Channel: 700 - Brightness:65; Contrast:85; Sensitivity:6;
Image: E:\Stefan\2008\07\040708 Prot Gel CXCL13 digest timecourse\..\Time course CXCL13 digest_1_700.TIF
Remarks:

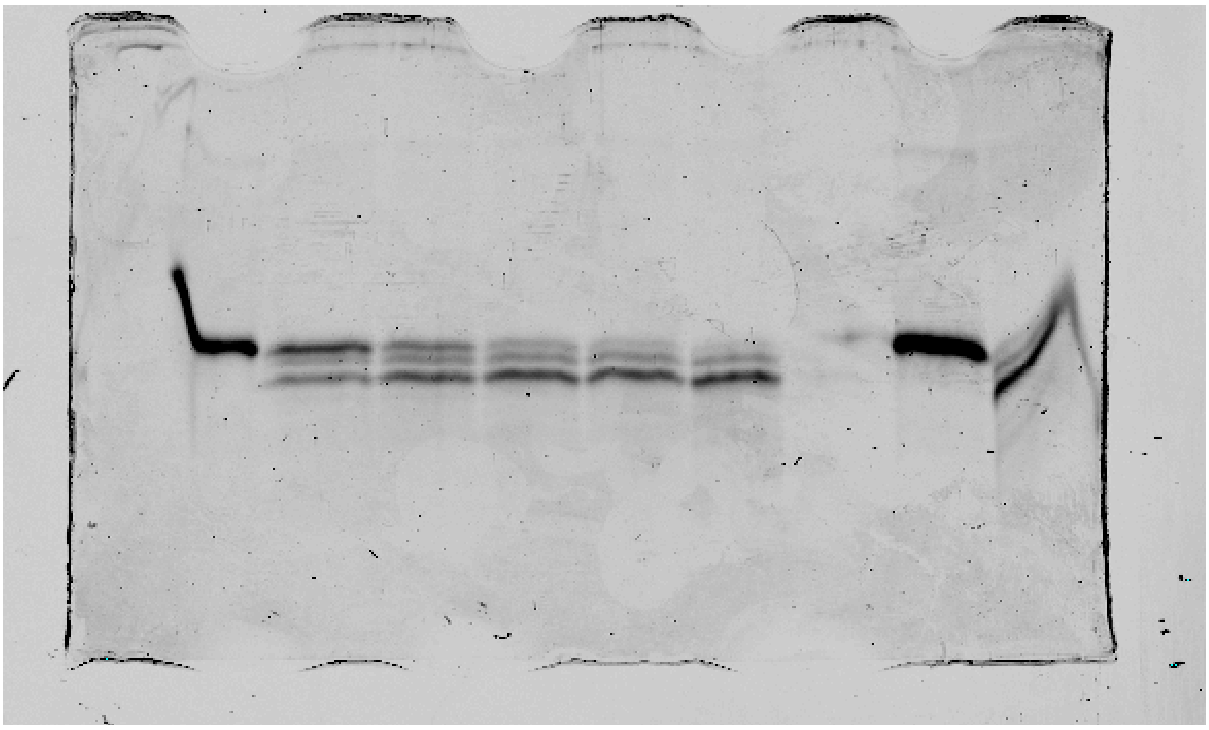

Supplement: Supplementary file 4 — Source Data [file 41467_2020_17135_MOESM4_ESM.zip › main figures/Fig 5A_040708_1.pdf]
