# Supplementary material for: B cell zone reticular cell microenvironments shape CXCL13 gradient formation
Source: Nat Commun. 2020 Jul 22;11:3677. doi: 10.1038/s41467-020-17135-2 (PMC7376062; doi:10.1038/s41467-020-17135-2)

RP HPLC Fractons Gel 2
Wed Dec 11 16:35:04 CET 2019
Project: 050309 CXCL13 digest in presence of GAGs
Scan: CXCL13 cathB digest plus GAGs

Channel: 700 - Brightness:33; Contrast:54; Sensitivity:7;
Image: E:\Stefan\2009\03\050309 CXCL13 digest in presence of GAGs\..\RP HPLC Fractons Gel 2_700.TIF
Remarks: Analysis derived from "CXCL13 cathB digest GAGs" using the following operations: 1. Median

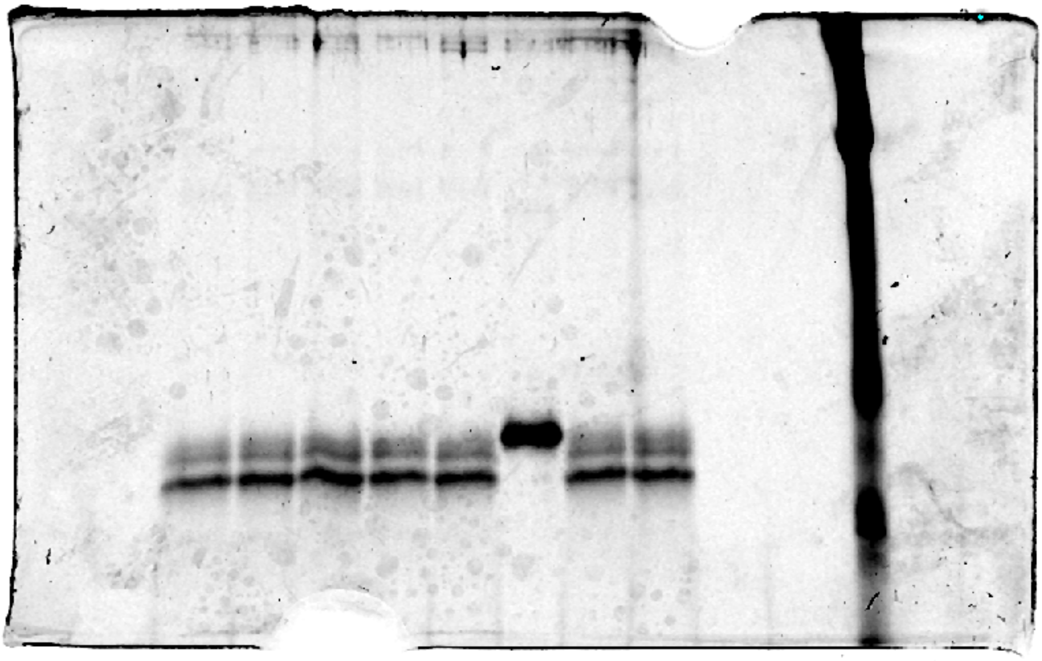

Supplement: Supplementary file 4 — Source Data [file 41467_2020_17135_MOESM4_ESM.zip › main figures/Fig 5C_050309.pdf]
