# Supplementary material for: B cell zone reticular cell microenvironments shape CXCL13 gradient formation
Source: Nat Commun. 2020 Jul 22;11:3677. doi: 10.1038/s41467-020-17135-2 (PMC7376062; doi:10.1038/s41467-020-17135-2)

2608_1
Wed Dec 11 16:22:07 CET 2019
Project: CXCL13 cathB digest different pH 261108
Scan: CXCL13cathB digest

Channel: 700 - Brightness:50; Contrast:57; Sensitivity:8;
Image: E:\Stefan\2008\11\CXCL13 cathB digest different pH 261108\CXCL13cathB digest\2508_700.TIF
Remarks:

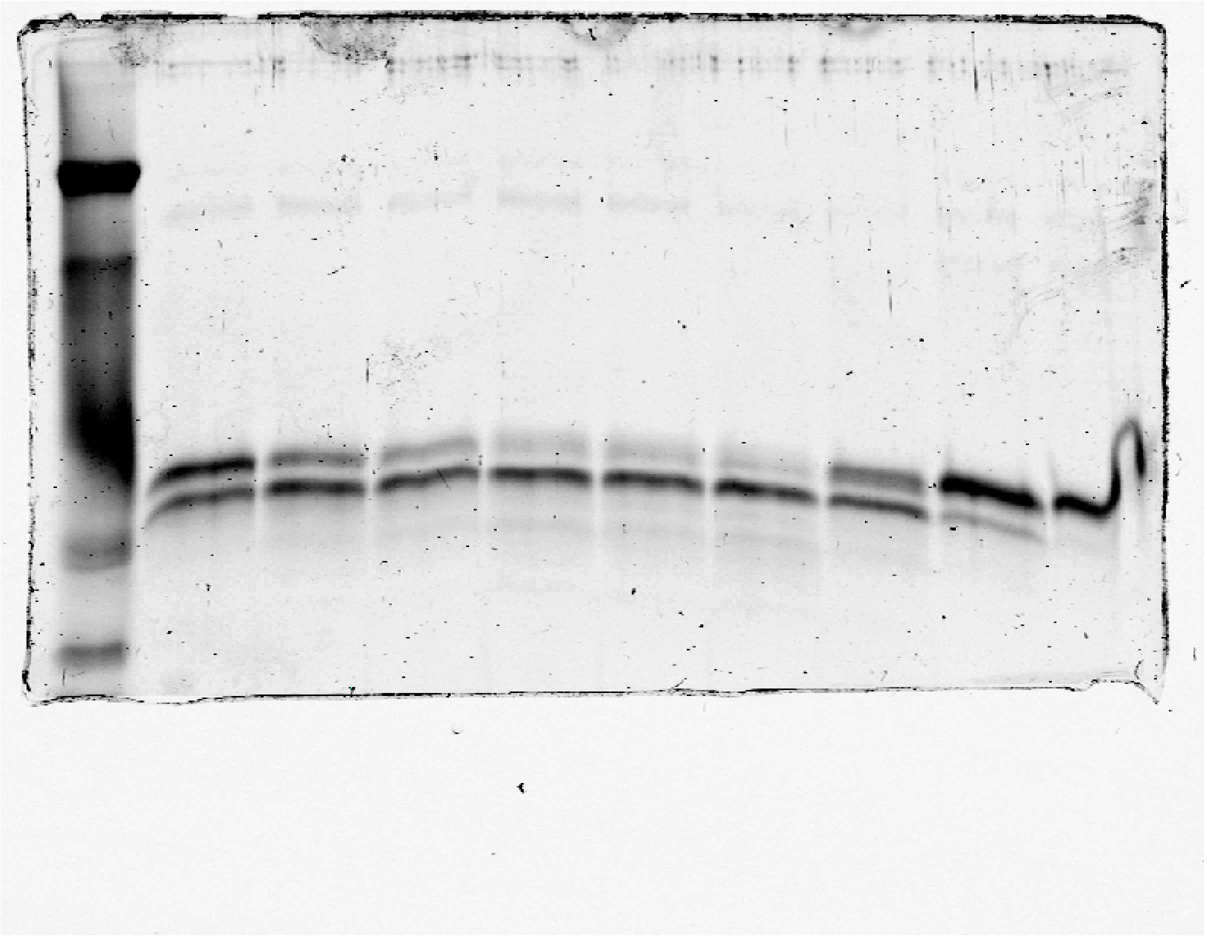

Supplement: Supplementary file 4 — Source Data [file 41467_2020_17135_MOESM4_ESM.zip › supplementary figures/Fig S4B.pdf]
