# Supplementary figures and images for: B cell zone reticular cell microenvironments shape CXCL13 gradient formation
Source: Nat Commun. 2020 Jul 22;11:3677. doi: 10.1038/s41467-020-17135-2 (PMC7376062; doi:10.1038/s41467-020-17135-2)

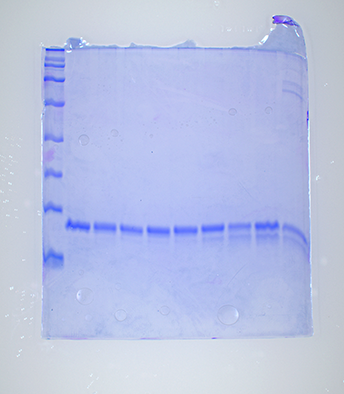

Supplement: Supplementary file 4 — Source Data [file 41467_2020_17135_MOESM4_ESM.zip › supplementary figures/S4a.tif]
